# Chimerization at the *AQP2–AQP3* locus is the genetic basis of melarsoprol–pentamidine cross-resistance in clinical *Trypanosoma brucei gambiense* isolates

**DOI:** 10.1016/j.ijpddr.2015.04.002

**Published:** 2015-05-07

**Authors:** Fabrice E. Graf, Nicola Baker, Jane C. Munday, Harry P. de Koning, David Horn, Pascal Mäser

**Affiliations:** a Swiss Tropical and Public Health Institute, CH-4051 Basel, Switzerland; b University of Basel, CH-4000 Basel, Switzerland; c Biological Chemistry & Drug Discovery, College of Life Sciences, University of Dundee, Dow Street, Dundee DD1 5EH, UK; d Institute of Infection, Immunity and Inflammation, University of Glasgow, Glasgow, G12 8TA, UK

**Keywords:** Human African trypanosomiasis, Sleeping sickness, *Trypanosoma brucei gambiense*, Drug resistance, Melarsoprol, Pentamidine, Aquaporin, Reverse genetics

## Abstract

Aquaglyceroporin-2 is a known determinant of melarsoprol–pentamidine cross-resistance in *Trypanosoma brucei brucei* laboratory strains. Recently, chimerization at the *AQP2–AQP3* tandem locus was described from melarsoprol–pentamidine cross-resistant *Trypanosoma brucei gambiense* isolates from sleeping sickness patients in the Democratic Republic of the Congo. Here, we demonstrate that reintroduction of wild-type *AQP2* into one of these isolates fully restores drug susceptibility while expression of the chimeric *AQP2/3* gene in *aqp2–aqp3* null *T.* *b.* *brucei* does not. This proves that *AQP2–AQP3* chimerization is the cause of melarsoprol–pentamidine cross-resistance in the *T.* *b.* *gambiense* isolates.

## Highlights

• Expression of *AQP2* restores drug susceptibility in a resistant *Trypanosoma brucei gambiense* isolate.
• The *AQP2/3* chimera from the resistant isolate does not complement *AQP2* deletion.
• Hence *AQP2/3* chimerization accompanied by loss of *AQP2* is the cause of drug resistance.

## Graphical Abstract

**Figure:**
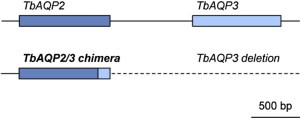

## 1 Introduction

*Trypanosoma brucei gambiense* is the causative agent of West-African sleeping sickness and responsible for 98% of today's cases of human African trypanosomiasis (HAT) (Brun et al., 2010). HAT is a fatal disease whose treatment exclusively relies on chemotherapy. Only five drugs are available: suramin and pentamidine for the first, haemolymphatic stage of the disease, melarsoprol and nifurtimox/eflornithine combination therapy for the second stage, when the parasites have infested the central nervous system. These drugs cause severe side effects and are difficult to administer (Brun et al., 2010). New drug candidates are in clinical development (Mäser et al., 2012), but until they are available for treatment, the current drugs must be used sustainably. Therefore understanding the molecular mechanism of drug resistance is a prerequisite. Drug resistance studies with *Trypanosoma brucei brucei* lab strains have identified loss of drug uptake as the major mechanism of drug resistance in trypanosomes. This is due to mutations in the transporters responsible for drug uptake. The clinical drugs melarsoprol and pentamidine share two common transporter systems, the adenosine transporter 1 (TbAT1, also called P2; Carter and Fairlamb, 1993; Carter et al., 1995; Mäser et al., 1999) and aquaglyceroporin 2 (Baker et al., 2012). Genetic knock-out of either transporter gene, but particularly of *AQP2*, led to melarsoprol–pentamidine cross-resistance (MPXR) (Matovu et al., 2003; Baker et al., 2012, 2013).

Drug resistance of *T.* *b.* *gambiense* in the field has been controversial. The occurrence of mutant *TbAT1* alleles correlated to some extent with melarsoprol treatment failures (Matovu et al., 2001; Maina et al., 2007; Kazibwe et al., 2009), but no unambiguous genetic marker for resistance has been established so far. Recently, mutations at the *AQP2–AQP3* (Tb927.10.14170/Tb927.10.14160) tandem locus were found in *T.* *b.* *gambiense* isolates from the Democratic Republic of the Congo (Graf et al., 2013). In particular, a set of 41 isolates from Mbuji-Mayi, a HAT focus of exceptionally high melarsoprol treatment failure rates (Pyana et al., 2014), all carried a chimeric aquaglyceroporin, presumably formed by homologous recombination between *AQP2* and *AQP3*; a putative single-strand annealing mechanism accompanied by deletion of segments of *AQP2* and *AQP3* (Graf et al., 2013). The *AQP2/3_(814_*_)_ chimera, the first 813 b derived from *AQP2* and the last 126 b from *AQP3*, was in-frame, transcribed and homozygous. A second *AQP2/3* chimeric gene, *AQP2/3_(880)_* with just the last 60 bp derived from *AQP3*, had been described in the *T.* *b.* *gambiense* isolates from Mbuji-Mayi (Pyana et al., 2014). In the present study we did not detect the *AQP2/3_(880)_* gene either with direct sequencing of PCR products or after cloning of the PCR products into expression vectors. The isolates carrying the chimeric gene exhibited a markedly decreased melarsoprol sensitivity *in vivo* (Pyana et al., 2014). Those that were tested *in vitro* were cross-resistant to melarsoprol and pentamidine; to our knowledge, the first example of MPXR from clinical *T.* *b.* *gambiense* isolates (Graf et al., 2013).

Thus, the important question remained: Is the MPXR phenotype of the *T.* *b.* *gambiense* isolates from Mbuji-Mayi caused by the observed chimerization at the *AQP2–AQP3* locus? And if so, is it the presence of the *AQP2/3_(814)_* chimera or the absence of wild-type *AQP2* that causes drug resistance? Here, we answer these questions by (i) re-introducing wild-type *AQP2* into one of the mutant *T.* *b.* *gambiense* isolates and (ii) expressing the chimeric *AQP2/3_(814)_* gene from *T.* *b.* *gambiense* in *T.* *b.* *brucei*.

## 2 Materials and methods

### 2.1 Cell lines, cell culture and *in vitro* drug sensitivity assay

*T.* *b.* *brucei* 2T1 cells (Alsford et al., 2005) and 2T1 *aqp2*–*aqp3* double knock-out cells (Alsford et al., 2012) were maintained in HMI-11 medium. Puromycin (0.2 µg/ml) and phleomycin (0.5 µg/ml) were added for 2T1 cells. For 2T1 *aqp2–aqp3* double knock-out cells blasticidin (10 µg/ml) and G418 (2 µg/ml) were added in addition. Hygromycin (2.5 µg/ml), instead of puromycin, was added after transfection with chimeric *AQP2/3_(814)_*. *T.* *b.* *gambiense* 40AT (MHOM/CD/INRB/2006/07; Pyana et al., 2011) were cultured in HMI-9 medium with 15% FCS and 5% human serum, plus blasticidin (5 µg/ml) after transfection. *In vitro* drug sensitivities were determined as described (Graf et al., 2013). For the inducible cells, 1 µg/ml tetracycline (tet) was added 24 h prior to the assay.

### 2.2 Plasmids and transfection

The chimeric *AQP2/3_(814)_* gene (GenBank accession KF564935) was amplified by PCR with primers AQP_HindIII_F (ccgcaagcttatgcagagccaaccagac) and AQP_BamH1_R (ccgcggatccttagtgtggcacaaaatatt), or AQP_Xba1_F (ccgctctagaatgcagagccaaccagac) and AQP_BamH1_R, and cloned into the pRPa-series of tetracycline-inducible expression vectors (http://www.lifesci.dundee.ac.uk/groups/david-horn/resources). Vector inserts were checked for fidelity by Sanger sequencing (Microsynth). Bloodstream-form *T.* *b.* *brucei* were transfected as previously described (Baker et al., 2012). Clones were obtained by limiting dilution in standard HMI-11 medium plus antibiotics (see above). The *AQP2* gene was amplified from wild-type *T.* *brucei* 427 parasites and the *AQP2/3_(569–841)_* gene from the derived, pentamidine-resistant, strain B48, using proof-reading polymerase and oligonucleotides which added an ApaI site to the 5′ end and a BamHI site to the 3′ end of the genes. The genes were ligated into pGEM-T Easy vector, and digested out using the added restriction sites. They were then ligated into similarly digested pHD1336 vector, to give plasmids pHDK21 (*AQP2*) and pHDK34 (*AQP2/3_(569–841)_*). Both plasmids were checked by Sanger Sequencing (Eurofins MWG Operon). Bloodstream-form *T.* *b.* *gambiense* were transfected with pHDK21 and pHDK34 as follows: 4 × 10^7^ cells were resuspended in 100 µl Tb-BSF nucleofection buffer (Schumann Burkard et al., 2011) (90 mM NaHPO_3_, 5 mM KCl, 0.15 mM CaCl_2_, 50 mM HEPES, pH 7.3) including 10 µg linearized plasmid DNA and placed in the nucleofection cuvette in the Amaxa Nucleofector (Lonza). Cells were electroporated using the program Z-001 and immediately transferred into 25 ml of pre-warmed HMI-9 medium containing 15% FCS, 5% human serum, and 20% sterile-filtered conditioned medium. Stable clones were obtained by limiting dilution and blasticidin selection (5 µg/ml). Correct integration was assessed by PCR on genomic DNA with primers AQP2_int_F (gtattggtgtggctgtcacg), AQP3_int_R (cccgttgagtaaccgatgtt), pAQP_F (aacacaccggtaccgtcatt) and pAQP_R (cttctcttgtgcgctgtacg).

Western blots of GFP-AQP2/3_(814)_ in 2T1 *aqp2–aqp3* null cells were performed as described (Baker et al., 2012). Western blots with GFP-AQP2/3_(814)_ in 2T1 wild-type cells were performed as follows: cells were lysed in NUPAGE® LDS sample buffer (Life Technologies), samples separated on precast 4–12% Bis-Tris Gradient Gels (NuPAGE Novex®, Life Technologies) and transferred to nitrocellulose membranes using the iBlot dry-blotting system (Novex®, Life Technologies) according to the manufacturer's recommendations. Western blots were developed with the ECL Western Blotting Substrate (Pierce) using a ChemiDoc™ MP Gel Imaging System (Biorad). Primary Antibody: rabbit anti-GFP (Abcam, Ab290); secondary antibody: goat anti-rabbit (SouthernBiotech, 4050-05).

## 3 Results and discussion

### 3.1 Expression of wild-type AQP2 re-sensitizes drug-resistant *T.* *b.* *gambiense*

**Fig. 1:**
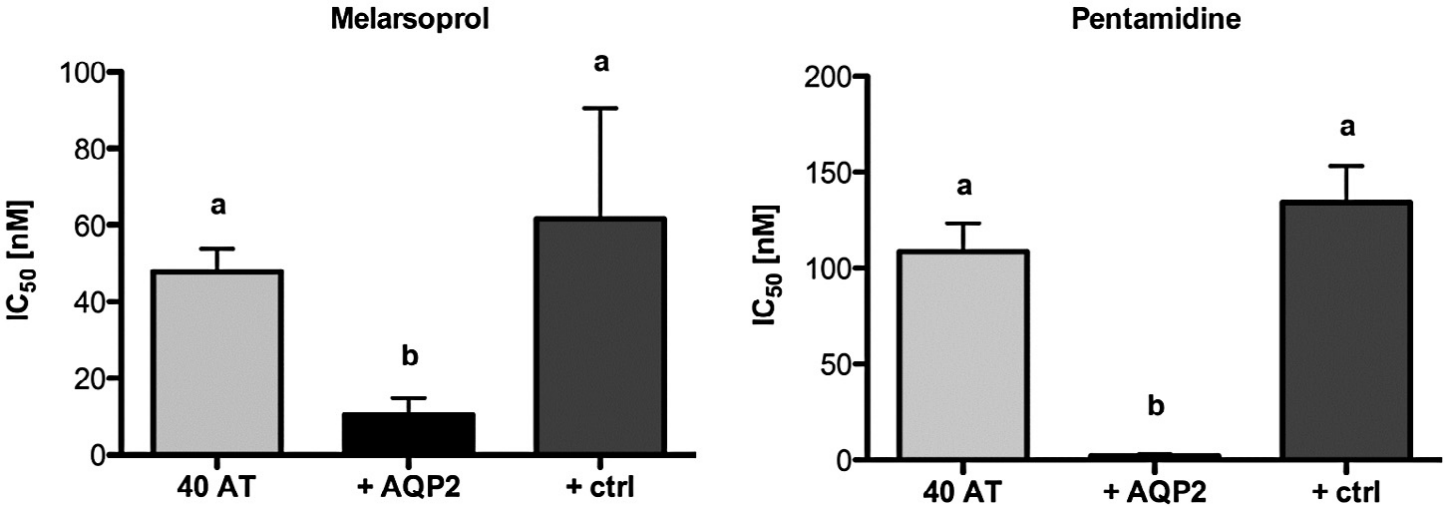
Introduction of *AQP2* into mutant *T.* *b.* *gambiense*. *In vitro* drug sensitivity of bloodstream-form *T.* *b.* *gambiense* 40AT (grey) transfected with *AQP2* (black) or dysfunctional *AQP2* (ctrl, dark grey). Error bars are standard errors of the mean. n = 6 independent experiments, each in duplicate. Small letters indicate significance groups as determined by one-way ANOVA and Tukey's post test using GraphPad Prism 5.0.

To test whether the lack of *bona fide AQP2* activity contributes to drug resistance in the isolates from Mbuji-Mayi, we introduced a ‘wild-type’ copy of *AQP2* into *T.* *b.* *gambiense* 40AT, isolated from a melarsoprol-relapse patient after treatment (Pyana et al., 2011). The gene was integrated into the highly transcribed *rRNA*-spacer locus. This shifted the IC_50_ of pentamidine from 108 nM to 2 nM and the IC_50_ of melarsoprol from 47 nM to 10 nM (Fig. 1), a level similar to the fully susceptible *T.* *b.* *gambiense* reference isolate STIB930 (which had an IC_50_ of 2 nM for pentamidine and 10 nM for melarsoprol; Graf et al., 2013). No shifts were observed with diminazene aceturate, a diamidine that is not an AQP2 substrate (Munday et al., 2014), or with phenylarsine oxide (data not shown), an arsenical that diffuses through the plasma membrane. The same results were obtained with three additional clones. As a negative control, we transfected the 40AT cells with a non-functional *AQP2* mutant from the MPXR *T.* *b.* *brucei* clone B48 (Munday et al., 2014). As expected, this did not affect susceptibility to melarsoprol or pentamidine (Fig. 1). These results demonstrate that *AQP2* is key to drug susceptibility in the MPXR *T.* *b.* *gambiense* isolate.

### 3.2 Expression of the chimeric AQP2/3_(814)_ in an aqp2–aqp3 null background

**Fig. 2:**
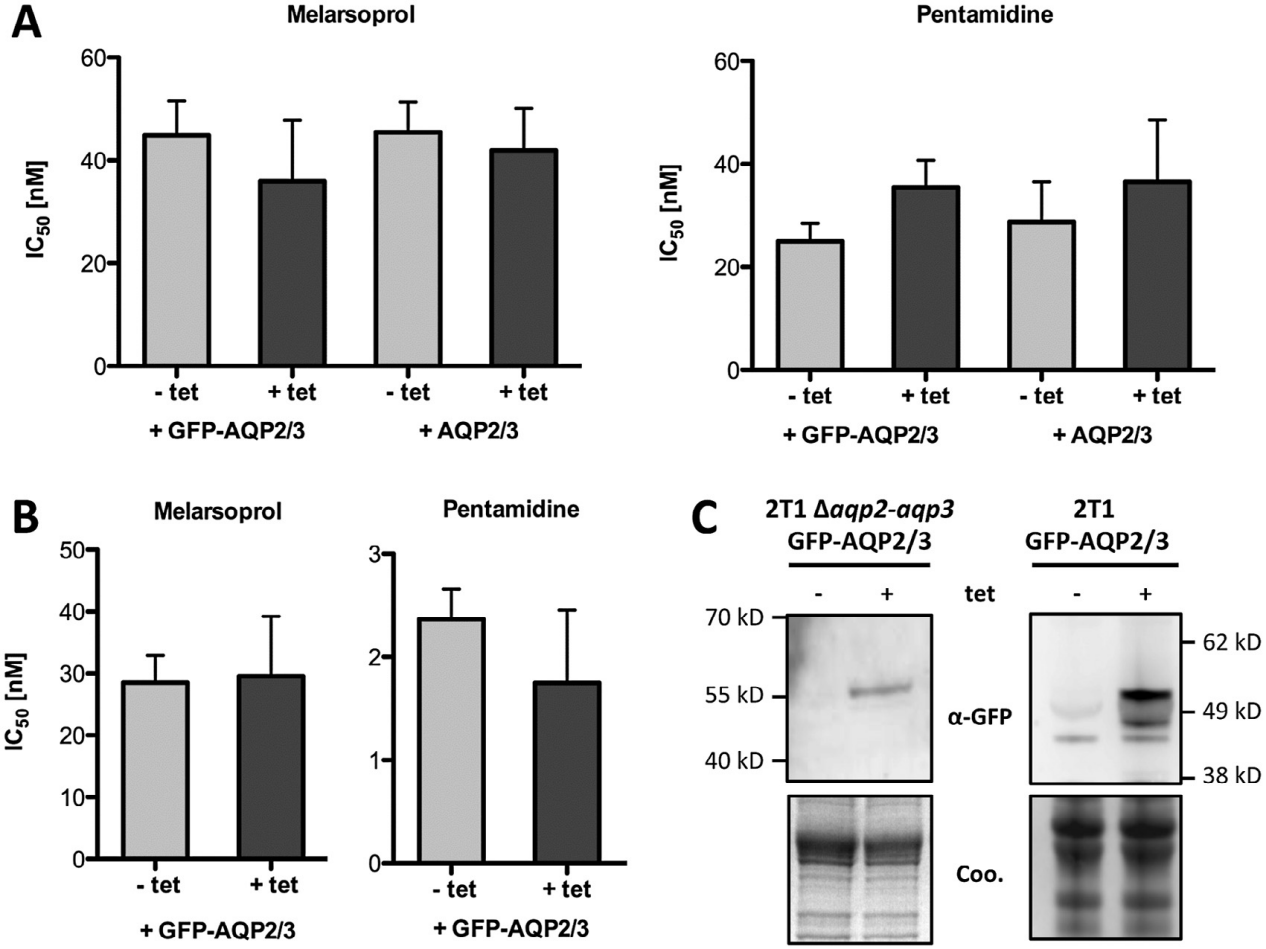
Expression of the *AQP2/3_(814)_* chimera in *T.* *b.* *brucei*. *In vitro* drug sensitivity of bloodstream-form *T.* *b.* *brucei* 2T1 *aqp2–aqp3* double null mutants (A) and parental 2T1 cells (B) transfected with a tetracycline (tet) inducible *AQP2/3_(814)_* chimera. Dark bars, tet (1 µg/ml) was added 24 h prior to the drug assay. Error bars are standard error of the mean. n = 4–5 independent experiments, each in duplicate. (C) Western blot with anti-GFP antibody demonstrating inducible expression of GFP-tagged *AQP2/3_(814)_* (Coo, Coomassie stain). The GFP-AQP2/3_(814)_ fusion proteins ran below their predicted molecular mass (approximately 60 kDa), which often applies for proteins with many transmembrane domains. The lower of the inducible bands in the blot on the right may represent unprocessed (e.g. unglyosylated) GFP-AQP2/3.

To test whether the chimeric AQP2/3*_(814)_* can complement AQP2 function with regard to drug uptake, we stably integrated the chimeric *AQP2/3_(814)_* gene from *T.* *b.* *gambiense* 40AT, either untagged or GFP-tagged, under the control of the tetracycline operator in a *T.* *b.* *brucei* host strain that expressed the tet repressor, and that carried a complete deletion of the *AQP2*–*AQP3* locus (Alsford et al., 2011). Tetracycline-inducible (1 µg/ml) expression of the chimeric AQP2/3_(814)_ protein was confirmed by immuno-fluorescence microscopy (data not shown) and by Western blotting with an anti-GFP antibody (Fig. 2C). Drug sensitivities were determined *in vitro* for melarsoprol and pentamidine. None of the transfected cell lines showed a significant difference in IC_50_ to pentamidine or melarsoprol when expression of *AQP2/3_(814)_* had been induced with tetracycline as compared to non-induced cells (Fig. 2A). This held true irrespective of the presence of the GFP tag. Thus no potential function in drug susceptibility could be attributed to the AQP2/3 chimera. Expression of ‘wild-type’ *AQP2* using the same over-expression system (untagged and GFP-tagged) did not just reverse MPXR but actually hypersensitized the *aqp2–aqp3* double null *T.* *b.* *brucei* to pentamidine and melarsoprol (Baker et al., 2012).

### 3.3 Expression of chimeric AQP2/3_(814)_ in wild-type cells does not affect drug sensitivity

Aquaporins form homotetramers where each monomer constitutes a single pore. Work on human aquaporins involved in diabetes insipidus has revealed that the expression of a mutant aquaporin can give rise to dominant negative effects (Mulders et al., 1998). To test for negative interactions of AQP2/3_(814)_ with ‘wild-type’ AQP2, the chimera was expressed in parental *T.* *b.* *brucei* 2T1 cells. The same tetracycline-inducible over-expression system was used. Again, no significant difference was observed regarding sensitivity to pentamidine and melarsoprol in tetracycline-induced versus uninduced cells (Fig. 2B). Hence, the AQP2/3_(814)_ chimera does not interfere with endogenous AQP2 function in *T.* *b.* *brucei* bloodstream-form cells.

## 4 Conclusion

Previous work on the correlation of occurrence of the chimeric *AQP2/3_(814)_* gene in *T.* *b.* *gambiense* isolates from the DRC with *in vitro* drug sensitivity (Graf et al., 2013) suggested a functional link between the chimera and MPXR. However, proof of a causal relationship was lacking. The AQP2/3_(814)_ chimeric protein consists mostly of AQP2 sequence, including the atypical second filter sequence (Baker et al., 2012). Overall, AQP2/3_(814)_ of the *T.* *b.* *gambiense* from Mbuji-Mayi has only 9 amino acid differences with AQP2. Moreover, the different *T.* *b.* *gambiense* isolates that harboured the chimeric gene were probably of clonal origin (Pyana et al., 2015) and may therefore not count as independent samples for the correlation of *AQP2/3_(814)_* genotype to MPXR phenotype. Thus reverse genetic engineering of bloodstream-form trypanosomes was required to establish a direct link between chimerization at the *AQP2–AQP3* locus in *T.* *b.* *gambiense* isolates and MPXR. This was only feasible because some of the isolates from Mbuji-Mayi had been adapted to axenic growth *in vitro* (Pyana et al., 2011).

The MPXR *T.* *b.* *gambiense* isolate 40AT was completely re-sensitized to melarsoprol and pentamidine when transfected with a wild-type copy of *AQP2*. This proves that the observed chimerization at the *AQP2–AQP3* locus is indeed the genetic basis of MPXR. The AQP2/3_(814)_ chimeric protein did not exhibit any role in conferring drug sensitivity when over-expressed in *T.* *b.* *brucei*, neither in a *aqp2–aqp3* null background nor in the *AQP2–AQP3* wild-type background. This further demonstrates that it is the absence of ‘wild-type’ AQP2, and not the presence of the AQP2/3_(814)_ chimera, that causes the MPXR phenotype. Deletion-based gene-fusion at the *AQP2–AQP3* locus by homologous recombination is likely facilitated by the high degree of sequence identity between *AQP2* and *AQP3*. Taken together, our findings strongly indicate that chimerization at the *AQP2–AQP3* locus causes melarsoprol–pentamidine cross-resistance in *T.* *b.* *gambiense*. This consequently increases the risk of treatment failures in problematic HAT foci such as Mbuji-Mayi of the Democratic Republic of the Congo. This is the first example where a genetic basis for drug-resistant sleeping sickness has been confirmed.

## Conflict of interest

The authors declared that there is no conflict of interest.
